# Reasons why Chinese smokers prefer not to use electronic cigarettes

**DOI:** 10.18332/tid/130477

**Published:** 2020-12-09

**Authors:** Zongshuan Duan, Yu Wang, Jidong Huang, Pamela B. Redmon, Michael P. Eriksen

**Affiliations:** 1 School of Public Health, Georgia State University, Atlanta, United States; 2 Emory Global Health Institute, Emory University, Atlanta, United States

**Keywords:** e-cigarettes, Chinese adult smokers, reasons not trying/using, smoking cessation

## Abstract

**INTRODUCTION:**

China is the world’s largest e-cigarette manufacturer. It also has the world’s largest smoking population. Although smoking is strongly associated with e-cigarette use, the prevalence of e-cigarette use is low among Chinese smokers compared with smokers in countries such as the US and UK. This study aims to explore the reasons why Chinese smokers prefer not to use e-cigarettes.

**METHODS:**

Cross-sectional data from the Tobacco Questions for Surveys (TQS) conducted in four large Chinese cities (Chengdu, Wuhan, Xiamen, and Xi’an) in 2017–2018 were analyzed. A multi-stage cluster sampling approach was applied to select a representative sample of adults for each city. Weighted percentages and 95% confidence intervals (CIs) were estimated for self-reported reasons why smokers in China had never tried e-cigarettes, in total and by demographic characteristics. Multivariate logistic regression models were used to examine the adjusted associations between the top reasons why smokers never tried e-cigarettes and demographic and socioeconomic characteristics.

**RESULTS:**

The top three reasons that Chinese adult smokers reported for never having tried e-cigarettes were: ‘I do not want to quit smoking’ (35.35%), ‘I do not think they would help me quit or cut down’ (24.31%), and ‘I am not addicted to smoking and don't need help to quit’ (14.93%). Other prominent reasons included: ‘I am concerned they are not safe enough’, and ‘I do not want to substitute one addiction for another’. Generally, there were no statistically significant associations between reasons why smokers never tried e-cigarettes and demographic and socioeconomic characteristics.

**CONCLUSIONS:**

Our results suggest that many Chinese smokers associate e-cigarette use with smoking cessation. Continued monitoring of smokers’ views, beliefs, and risk perceptions regarding e-cigarettes is warranted. Health education campaigns communicating the risks of e-cigarettes are also needed.

## INTRODUCTION

Tobacco and nicotine product markets have been substantially transformed by the emergence of electronic cigarettes (e-cigarettes) in the past decade in many countries^1-3^. E-cigarettes, also known as ‘electronic vaping products’ or ‘electronic nicotine delivery systems (ENDS)’, are ‘a diverse group of products that produce a heated aerosol, typically containing nicotine, which users inhale via a mouthpiece’^4^. Besides nicotine, the aerosol produced by e-cigarettes also contains other toxic substances that are harmful to consumers’ health^4-8^. Although the long-term health effects of e-cigarettes are still unknown, current evidence shows that e-cigarettes have an adverse effect on the function of the users’ respiratory system, cardiovascular system, endocrine system, etc.^4-8^.

Previous studies indicated that the prevalence of e-cigarette use was higher among current cigarette smokers than among non-smokers^1-3^. In the US, 49.4% of current cigarette smokers had ever used e-cigarettes, while only 6.5% of never cigarette smokers had ever used e-cigarettes, among adults in 2018^3^. Similar patterns were also observed in the UK^1^. The most common reason to use e-cigarettes reported by smokers was to help them quit smoking^9^, despite that the scientific evidence on the effectiveness of e-cigarettes as a cessation tool is still equivocal^10,11^.

With more than 300 million smokers, China is the world’s largest tobacco producer and consumer^12^. Although the prevalence of tobacco smoking has shown a slight decline in China from 28.1% in 2010 to 26.6% in 2018, smoking remains the leading preventable cause of death and illness^12-14^. China is also the world’s largest e-cigarette manufacturing base^15^. There are still no comprehensive national standards regulating e-cigarette manufacturing, sales, packaging, and advertising in China^16^. The 2018 China Global Adult Tobacco Survey (GATS) reported that 48.5% had heard of, 5.0% had ever used, and 0.9% were currently using e-cigarettes among Chinese adults aged ≥15 years^14^. The awareness and use of e-cigarettes varied across regions and population subgroups characterized by sociodemographic factors and smoking status^16-20^. Similar to what has been reported in the US and UK, e-cigarette use was also higher among smokers than among non-smokers in China; however, the prevalence of e-cigarette use among Chinese smokers was much lower^1,3,16,17^. While only about one-third to a half of the current smokers had never tried e-cigarettes in the US and UK, more than 80% of current adult smokers had never tried e-cigarettes in China^1,3,16,17^.

Given the significant differences in the patterns of e-cigarette use among smokers between China and other countries, it is important to understand what factors contributed to the low usage of e-cigarettes among Chinese smokers. Previous studies conducted in the US and UK provided a starting point for understanding the reasons why smokers never tried e-cigarettes^1,21^. One study used nationally representative data of US adult current smokers from 2017 to 2018 and found that the most commonly reported reasons for never having tried e-cigarettes included: ‘not wanting to substitute one addiction for another’, ‘concerns about their safety’, and ‘skepticism that e-cigarettes could not help them quit smoking’^21^. Another study using national representative data of the UK in 2019 found that the most commonly reported reasons for current smokers not trying e-cigarettes were: ‘I do not want to substitute one addiction to another’, ‘I am not addicted to smoking and don't need help to quit’, and ‘I do not know enough about them’^1^. Unlike in the US and UK, cigarette affordability is high and the intention to quit among smokers is low in China^14,22,23^. Consequently, Chinese smokers may have different reasons and/or concerns for not trying or using e-cigarettes. However, studies aimed at examining perceptions on e-cigarettes among smokers are scarce in China. Specifically, to our knowledge, there are no studies examining the reasons why smokers do not try or use e-cigarettes in China.

To better understand the perceptions of Chinese smokers on e-cigarettes to inform future research and policymaking in China, this study examines the reasons why such a large proportion of smokers had never tried e-cigarettes in China. This study also explores whether the reasons differ by sociodemographic characteristics.

## METHODS

### Study design, setting, participants

The data used in this study were obtained from the China Tobacco Questions for Surveys (TQS) study conducted from November 2017 to March 2018 in four large Chinese cities: Chengdu, Wuhan, Xiamen, and Xi’an. TQS contains a subset of key questions from the GATS on participants’ sociodemographic characteristics, usage of combustible tobacco products and e-cigarettes, secondhand smoke exposure, smoking cessation, etc.^24^. The TQS survey in each city was approved by its municipal health department Institutional Review Boards (IRB). All participants signed the informed consent form before information collection. Secondary data analysis of the de-identified TQS dataset was also approved by the IRB (IRB Number: H18183, Reference Number: 346974).

**Figure 1:**
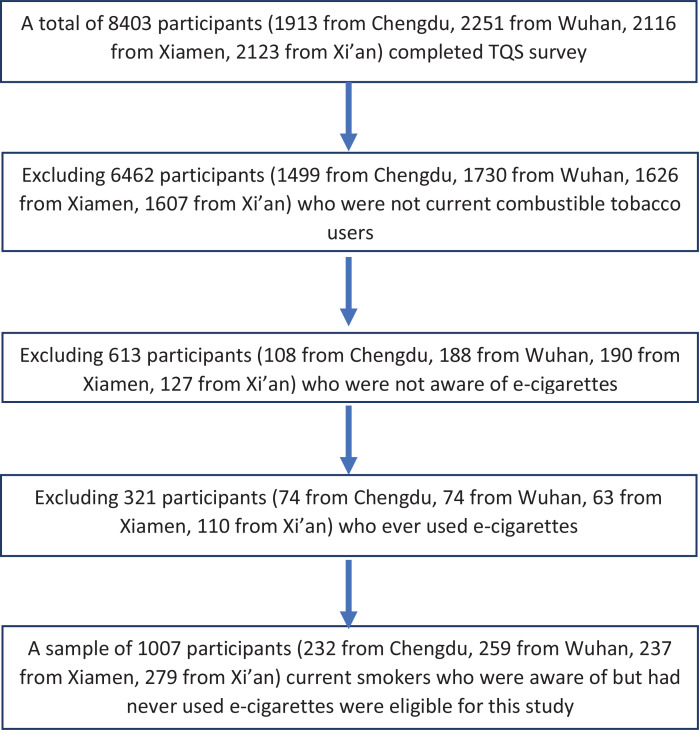
Flow chart for participants selected in the final analyses

The target population of TQS was non-institutionalized urban residents aged ≥15 years at the time of survey. Multi-stage cluster sampling was applied to generate a citywide representative sample for each city following the guidelines of the Sample Design Manual of GATS^25^. Detailed study design and sampling methods can be found in a previously published study using the TQS data^17^. A total of 8043 participants completed the TQS survey in all four cities. Only current smokers who were aware of, but had never used e-cigarettes, were eligible for this study. There were 6462 participants excluded because they were not current smokers, 613 participants excluded because they were not aware of any e-cigarette products, and 321 participants excluded because they had ever used e-cigarettes (Figure 1). This led to a total sample size of 1007, including 232 from Chengdu, 259 from Wuhan, 237 from Xiamen, and 279 from Xi’an.

### Measures and variables

Participants who reported smoking tobacco products (cigarettes, cigars, and pipes) daily or less than daily were categorized as current smokers. Participants were also asked whether they had ever heard of any type of e-cigarette products and whether they had ever used any type of e-cigarette products. Only current tobacco smokers who had heard of but never used e-cigarettes were asked about the reasons why they never tried e-cigarettes. The response categories included: ‘They are too difficult to get hold of’, ‘I am using other things to help me quit smoking’, ‘I would be embarrassed to use them in public’, ‘There are too many products to choose from’, ‘They cost too much’, ‘I do not like the way they look’, ‘I am not addicted to smoking and don't need help to quit’, ‘I do not want to quit smoking’, ‘I do not think they would help me to quit or cut down’, ‘I do not want to substitute one addiction for another’, and ‘I am concerned they are not safe enough’. Respondents were asked to answer ‘Yes’ or ‘No’ to each reason, and multiple ‘Yes’ selections were allowed.

The measure used in this study to capture the reasons why smokers never tried e-cigarettes was constructed using a combination of two methods. First, using a community based participatory research approach, a qualitative study was conducted, in collaboration with the China CDC in 2017, among a small group of Chinese adult smokers and medical doctors, to understand smokers’ opinions about e-cigarette use. The results from this qualitative study were the basis of the measure we used in this study. In addition, we refined our measure based on similar measures that were used in the studies conducted in the US^21^.

Demographic characteristics included respondents’ sex, age, education level, and current occupation. Age was grouped into: 15–24, 25–44, 45–64, and ≥65 years. Education level included: primary school completed or below, junior high school completed, senior high school completed, and college degree or above. Current occupation was categorized into three types: ‘government employee, teacher, or healthcare provider’; ‘factory, business, agriculture, or service industry employee’; and ‘not in the labor force’ which included the unemployed, students, homemakers, and retired. The categorizations were consistent with other national tobacco surveys in China, such as the China GATS^13,14^.

### Statistical analysis

Data analyses were conducted using SAS^®^ 9.4 (SAS Institute, Cary, North Carolina, US). The multi-stage sampling features were accounted for with survey procedures in SAS. Sample weights were calculated as the reciprocal of the selection probability and post-adjusted by gender and age groups in each city^25^. Answers like ‘I don't know’ or ‘Refused’ were coded as missing. There were less than 5% of participants with any missing value for variables of interest. We assumed that missing data arose completely at random and used pairwise deletion to handle missing data^26^. We estimated the percentages and 95% confidence intervals (CIs) of respondents who selected each reason for never having tried e-cigarettes in total and separately for each city. Furthermore, we report the percentages and 95% CIs for respondents who selected each reason by sex, age, education, and occupation, for the top five most selected reasons, for the four cities combined. Rao-Scott chi-squared tests were performed to check whether there were bivariate associations between sociodemographic characteristics and the top five selected reasons. Logistic regression models were used to examine the unadjusted and adjusted associations between the top five selected reasons and sociodemographic characteristics.

## RESULTS

### Demographic characteristics

**Figure 2:**
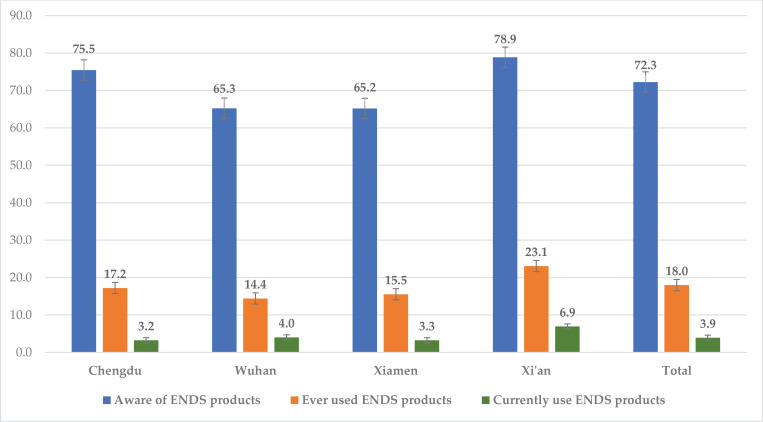
Percentage of adult smokers who were aware of e-cigarette products, ever used e-cigarette products, and currently use e-cigarette products in four Chinese Cities, 2018, TQS study (N=8403)

As illustrated in Figure 2, among current smokers residing in the four Chinese cities in our study, the overall prevalence of awareness of any e-cigarette product among adult smokers was 72.3% in 2018, with the highest in Xi’an (78.9%) and lowest in Xiamen (65.2%). In addition, the overall prevalence of ever using any type of ENDS product was 18.0% in 2018, ranging from 14.4% in Wuhan to 23.1% in Xi’an. Furthermore, among all current adult smokers from four cities, 3.9% reported currently using an e-cigarette product, ranging from 3.2% in Chengdu to 6.9% in Xi’an.

Only participants who were aware of but had never used e-cigarettes were included for further analysis in this study. In our study sample, more than 90% were men; about 45% were aged 25–44 years and about 35% were aged 45–64 years; more than 70% completed senior high school and more than one-third had a college degree or above; approximately 10% were government employees, teachers, or healthcare providers, and about half were factory, business, or service industry employees. The distributions of demographic characteristics overall and across the four cities are presented in Table 1.

**Table 1:** Demographic characteristics of adult smokers who had heard of but never used e-cigarettes in four Chinese cities, 2018

*Characteristics*	*Chengdu (N=232)*		*Wuhan (N=259)*		*Xiamen (N=237)*		*Xi'an (N=279)*		*Overall (N=1007)*	
	*n*	*%*	*n*	*%*	*n*	*%*	*n*	*%*	*n*	*%*
**Sex**										
Female	26	10.0	12	4.8	7	3.2	9	5.6	54	6.5
Male	206	90.0	247	95.2	230	96.8	270	94.4	953	93.5
**Age** (years)										
15–24	20	16.4	12	6.8	9	14.1	10	18.7	51	14.7
25–44	109	45.7	101	38.0	147	64.3	112	39.4	469	45.1
45–64	79	32.6	119	46.7	71	19.4	137	38.1	406	35.2
≥65	24	5.3	27	8.6	10	2.3	20	3.8	81	5.1
**Education level**										
Primary school or below	22	7.5	13	4.2	24	9.6	8	6.1	67	6.7
Junior high school completed	49	17.6	53	22.8	84	33.1	44	13.4	230	19.9
Senior high school completed	65	31.4	122	46.1	67	30.6	107	38.7	361	36.5
College degree or above	95	43.5	68	26.9	61	26.8	118	41.9	342	36.9
**Occupation**										
Gov. employee, teacher, healthcare provider	21	10.2	20	7.3	15	7.8	37	12.9	93	10.0
Factory, business, service industry employee	119	56.9	114	45.4	150	58.3	132	48.6	515	52.2
Not in the labor force^a^	90	32.9	124	47.3	71	34.0	107	38.5	392	37.8

a Respondents who were not in the labor force included students, homemakers, retired, and unemployed residents either able or unable to work.

### Reasons for never having tried e-cigarettes

In all four cities combined, the top three reasons that current smokers reported for never having tried e-cigarettes were: ‘I do not want to quit smoking’ (35.4%; 95% CI: 26.6–44.1), ‘I do not think they would help me to quit or cut down’ (24.3%; 95% CI: 18.7–29.9), and ‘I am not addicted to smoking and don't need help to quit’ (14.9%, 95% CI: 10.5–19.4). In addition, about 15% of respondents reported that ‘I am concerned they are not safe enough’ (95% CI: 10.7–19.1) and ‘I do not want to substitute one addiction for another’ (95% CI: 10.6–19.1). Approximately 12% of respondents reported ‘I do not like the way they look’ (95% CI: 9.5–14.9) and ‘They cost too much’ (95% CI: 8.6–15.8). Less than 10% of respondents reported that ‘There are too many products to choose from’ and ‘They are too difficult to get hold of’. Approximately 5% mentioned that ‘I would be embarrassed to use them in public’ and ‘I am using other things to help me quit smoking’ (Table 2).

**Table 2:** Percentages and 95% confidence intervals (CIs) of current adult smokers who had heard but never used e-cigarettes that reported a specific reason for never having tried e-cigarettes in four Chinese cities and overall, 2018

Reasons^*^	*Chengdu (N=232)*		*Wuhan (N=259)*		*Xiamen (N=237)*		*Xi'an (N=279)*		*Overall (N=1007)*	
	*%*	*95% CI*	*%*	*95% CI*	*%*	*95% CI*	*%*	*95% CI*	*%*	*95% CI*
A	40.2	23.0–57.5	42.4	26.6–58.1	26.1	15.3–36.9	29.9	18.1–41.7	35.4	26.6–44.1
B	13.1	9.2–16.9	29.4	15.7–43.1	24.0	12.6–35.3	32.8	19.6–46.0	24.3	18.7–29.9
C	8.6	3.4–13.8	17.7	7.5–27.9	19.5	9.2–29.9	17.4	9.9–24.9	14.9	10.5–19.4
D	6.2	2.4–9.9	17.2	7.2–27.2	11.2	5.1–17.3	24.8	15.6–33.9	14.9	10.7–19.1
E	7.1	3.7–10.5	22.9	9.4–36.3	8.3	2.2–14.5	20.8	11.2–30.5	14.8	10.6–19.1
F	7.9	3.7–12.2	17.4	9.0–25.9	7.9	2.5–13.3	15.6	10.6–20.5	12.2	9.5–14.9
G	8.1	0.4–15.9	14.5	4.6–24.3	6.3	2.1–10.4	17.8	11.0–24.6	12.2	8.6–15.8
H	8.2	0.4–16.1	9.0	2.3–15.7	3.4	1.1–5.8	13.9	8.3–19.4	9.3	5.6–13.1
I	7.7	0.1–15.2	12.0	4.0–20.1	5.1	0.7–9.5	6.5	2.7–10.4	7.9	4.6–11.2
J	0.9	0.0–2.2	6.6	2.3–10.9	3.2	0.0–7.0	11.3	6.0–16.6	5.6	3.5–7.7
K	1.9	0.0–3.7	3.3	0.1–6.4	3.8	0.0–8.1	9.6	4.5–14.8	4.8	2.9–6.7

* A: I do not want to quit smoking; B: I do not think they would help me to quit or cut down; C: I am not addicted to smoking and don’t need help to quit; D: I am concerned they are not safe enough; E: I do not want to substitute one addiction for another; F: I do not like the way they look; G: They cost too much; H: There are too many products to choose from; I: They are too difficult to get hold of; J: I would be embarrassed to use them in public; K: I am using other things to help me quit smoking.

The top three reasons for never having tried e-cigarettes for Chengdu and Xiamen were the same as in all four cities combined. In Wuhan and Xi’an, ‘I am not addicted to smoking and don't need help to quit’ was not one of the top three reasons. Instead, in Wuhan, 22.9% (95% CI: 9.4– 36.3) of respondents selected ‘I do not want to substitute one addiction for another’ and in Xi’an, 24.8% (95% CI: 15.6–33.9) of respondents selected ‘I am concerned they are not safe enough’ as a reason for never having tried e-cigarettes (Table 2).

### Bivariate associations between the top five reasons and sociodemographic characteristics

Generally, female respondents were more likely to select each reason than male respondents, however, the associations were not statistically significant due to the small sample size of women in our study. Compared to respondents not in the labor force, government employees, teachers, and healthcare providers were less likely to select ‘I do not want to quit smoking’ as a reason for never having tried e-cigarettes [(26.0%; 95% CI: 15.6–36.4) vs (40.3%; 95% CI: 31.3–49.3); p=0.04]. Respondents with different sociodemographic characteristics, including sex, age, education, and occupation, did not differ significantly in selecting ‘I don't think they would help me to quit or cut down’, ‘I am concerned they are not safe enough’, and ‘I do not want to substitute one addiction for another’, as reasons for never having tried e-cigarettes.

There were some statistically significant bivariate associations between sociodemographic characteristics and selecting ‘I am not addicted to smoking and don't need help to quit’ as a reason for never having tried e-cigarettes. Respondents who were aged 25–44 years were more likely to select this reason than respondents who were aged 15–24 years [(18.7%; 95% CI: 13.1–24.3) vs (5.5%; 95% CI: 0.0–11.3); p=0.02] and respondents who were aged ≥65 years [(18.7%; 95% CI: 13.1–24.3) vs (8.7%; 95% CI: 2.9–14.5); p=0.02]. In addition, respondents with an education level of primary school completed or below were less likely to select this reason than respondents who completed junior high school [(5.9%; 95% CI: 0.8–11.1) vs (19.3%; 95% CI: 12.9–25.6); p=0.01] and respondents with a college degree or above [(5.9%; 95% CI: 0.8–11.1) vs (16.6%; 95% CI: 11.2–22.0); p=0.01]. Rao-Scott chi-squared tests also showed that there was an association between age group and selecting this reason (χ^2^=12.5, *df*=3, p=0.006) (Table 3).

**Table 3:** Percentages and 95% confidence intervals (CIs) of current adult smokers who had heard but never used e-cigarettes that reported a specific reason (top five reasons presented)^*^ for never having tried e-cigarettes in four Chinese cities overall by sociodemographic characteristics, 2018 (N=1007)

*Characteristics*	*Reason A*		*Reason B*		*Reason C*		*Reason D*		*Reason E*	
	*%*	*95% CI*	*%*	*95% CI*	*%*	*95% CI*	*%*	*95% CI*	*%*	*95% CI*
**Total**	35.4	26.6–44.1	24.3	18.7–29.9	14.9	10.5–19.4	14.9	10.7–19.1	14.8	10.6–19.1
**Sex**										
Female	50.0	28.7–71.2	31.9	8.2–55.5	15.1	4.7–25.5	27.6	1.8–53.4	21.4	0.0–47.9
Male	34.3	26.6–42.0	23.8	18.3–29.2	14.9	10.8–19.1	14.0	9.7–18.2	14.4	9.9–18.8
**Age** (years)										
15–24	31.4	12.3–50.5	17.8	2.9–32.7	5.5	0.0–11.3	8.2	0.0–21.8	10.5	0.0–24.3
25–44	35.0	26.2–43.7	24.5	17.8–31.2	18.7	13.1–24.3	17.1	11.4–22.9	15.0	9.6–20.4
45–64	37.1	28.7–45.5	27.7	21.3–34.0	15.1	9.6–20.5	15.7	10.4–21.0	17.0	10.5–23.4
>65	38.8	24.0–53.6	19.7	8.9–30.4	8.7	2.9–14.5	10.0	3.3–16.7	12.5	4.5–20.5
**Education level**										
Primary school or below	42.3	19.8–64.8	33.5	8.9–58.1	5.9	0.8–11.1	28.7	3.9–53.4	25.6	0.0–52.2
Junior high school completed	36.3	26.7–46.0	24.6	16.7–32.4	19.3	12.9–25.6	12.4	6.7–18.1	18.1	10.7–25.5
Senior high school completed	33.5	24.3–42.6	21.6	13.3–29.8	12.3	6.0–18.7	14.2	7.8–20.7	12.5	5.8–19.3
College degree or above	35.4	23.8–47.0	25.2	18.3–32.1	16.6	11.2–22.0	14.4	8.7–20.0	13.3	8.7–17.8
**Occupation**										
Gov. employee, teacher,healthcare provider	26.0	15.6–36.4	24.9	14.7–35.1	21.6	9.4–33.8	19.0	8.8–29.2	11.8	4.8–18.8
Factory, business, service industry employee	33.9	23.4–44.5	20.8	14.2–27.4	14.9	9.8–20.0	15.6	10.5–20.7	15.1	9.5–20.7
Not in the labor force	40.3	31.3–49.3	28.7	21.6–35.8	13.4	9.2–17.6	13.0	6.7–19.3	15.6	9.2–22.0

* A: I do not want to quit smoking; B: I do not think they would help me to quit or cut down; C: I am not addicted to smoking and don't need help to quit; D: I am concerned they are not safe enough; E: I do not want to substitute one addiction for another.

### Adjusted associations between the top five reasons and sociodemographic characteristics

Overall, most of the adjusted associations between demographic characteristics and reasons for not using e-cigarettes were not statistically significant (Table 4), with only a couple exceptions. Compared with respondents not in the labor force, government employees, teachers, and healthcare providers were less likely to choose ‘I do not want to quit smoking’ as a reason for not trying e-cigarettes (AOR=0.51; 95% CI: 0.28–0.93); factory, business, and service industry employees were less likely to choose ‘I do not think they would help me to quit or cut down’ as a reason for not trying e-cigarettes (AOR=0.62; 95% CI: 0.40–0.97), after adjusting for sex, age, and education (Table 4).

**Table 4:** Adjusted odds ratios (AORs)^a^ of reporting a specific reason (top five reasons presented)* for never having tried e-cigarettes among current adult smokers who had heard but never used e-cigarettes in four Chinese cities overall, 2018 (N=1007)

*Characteristics*	*Reason A*		*Reason B*		*Reason C*		*Reason D*		*Reason E*	
	*AOR*	*95% CI*	*AOR*	*95% CI*	*AOR*	*95% CI*	*AOR*	*95% CI*	*AOR*	*95 % CI*
**Sex**										
Female	1.8	0.9–3.7	1.5	0.6–3.6	1.5	0.7–3.3	2.7	0.9–7.9	1.6	0.5–5.2
Male	Ref.		Ref.		Ref.		Ref.		Ref.	
**Age** (years)										
15–24	0.9	0.3–2.1	1.1	0.3–3.7	0.6	0.1–2.2	0.8	0.1–6.4	1.1	0.2–6.2
25–44	1.1	0.6–2.1	2.0	0.9–4.9	2.1	0.9–5.2	2.4	0.7–7.7	1.8	0.6–5.3
45–64	1.1	0.6–2.2	2.2	0.9–5.0	1.6	0.6–4.1	2.1	0.7–6.6	1.9	0.6–5.7
≥65	Ref.		Ref.		Ref.		Ref.		Ref.	
**Education level**										
Primary school or below	1.0	0.4–2.4	1.3	0.5–3.5	0.4	0.2–1.1	3.3	0.8–13.1	2.4	0.6–10.2
Junior high school completed	0.9	0.5–1.6	0.8	0.5–1.5	1.3	0.8–2.4	1.0	0.5–2.0	1.4	0.7–2.7
Senior high school completed	0.8	0.5–1.3	0.7	0.4–1.2	0.8	0.4–1.6	1.1	0.6–2.2	0.9	0.5–1.8
College degree or above	Ref.		Ref.		Ref.		Ref.		Ref.	
**Occupation**										
Gov. employee, teacher, healthcare provider	0.5	0.3–0.9	0.7	0.4–1.2	1.6	0.8–3.5	1.9	0.7–4.7	0.8	0.4–1.8
Factory, business, service industry employee	0.8	0.5–1.3	0.6	0.4–1.0	1.0	0.6–1.7	1.4	0.9–2.4	1.1	0.7–1.8
Not in the labor force	Ref.		Ref.		Ref.		Ref.		Ref.	

a The logistic regression models were adjusted for sex, age, education level, and occupation type.

* A: I do not want to quit smoking; B: I do not think they would help me to quit or cut down; C: I am not addicted to smoking and don’t need help to quit; D: I am concerned they are not safe enough; E: I do not want to substitute one addiction for another.

## DISCUSSION

Our results showed that major reasons for not trying e-cigarettes among Chinese adult smokers included low intention to quit smoking, concerns about e-cigarettes’ effectiveness as a smoking cessation method, and belief of being able to quit any time without aid, as well as concerns about substitution with another addictive behavior, concerns about e-cigarettes’ safety, appearance, and cost. Specifically, more than one-third of smokers who have heard of e-cigarettes indicated that they never tried e-cigarettes because they did not want to quit, and about onefourth of smokers indicated that they were concerned about the products’ effectiveness to help quit smoking. In general, there were no consistent significant associations between respondents’ sociodemographic characteristics and the reasons they reported for never having tried e-cigarette products.

Top reasons why smokers in China had never tried e-cigarette products were similar to those reported in the US and UK. For example, ‘I do not want to substitute one addiction for another’ was one of the top reasons current smokers never tried e-cigarettes in all three countries (60% in the US, 16% in the UK, and 15% in China)^1,21^. However, there were differences in those top reasons across the three countries as well. For example, some reasons/concerns were more prominent among Chinese smokers. In our study, the most selected reason for never having tried e-cigarettes among Chinese smokers was that they did not want to quit smoking, which was likely attributable to the low intention to quit among Chinese smokers^14^. The 2018 China GATS reported that only 20% of current/former smokers made at least one quit attempt in the past 12 months, and only 16% of current smokers planned to quit or thought about quitting in the next 12 months in China^14^. However, 58% of current smokers reported intention to quit in the UK, and 55% of current smokers made at least one quit attempt in the past 12 months in the US in 2018^27,28^. More efforts to promote tobacco cessation are needed in China. In addition, although ‘I don’t think they would help me quit or cut down’ was the second most selected reason (24%) in China and the third most selected reason in the US (52%)^21^, it was not one of the top three reasons in the UK^1^. This could be due to different regulatory guidelines towards e-cigarettes across these three countries. In the US, e-cigarettes are considered as a tobacco product under the regulation of the Food and Drug Administration (FDA)^29^. In China, e-cigarettes are not categorized as either a tobacco product or a cessation aid^16^. In the UK, physicians were encouraged to promote e-cigarettes to current smokers who planned to quit as a cessation aid^30^.

About 12% of respondents selected ‘they (e-cigarettes) cost too much’ as a reason why they had never tried e-cigarettes, which indicated that cost was an important reason for not trying e-cigarettes among Chinese smokers. However, it is not one of the top five reasons in our study. One possible explanation could be that a significant proportion of smokers in China had no intention to try e-cigarettes, so they had not considered the price of e-cigarettes yet. This hypothesis was supported by our study results. In our study, the top three reasons for not trying e-cigarettes were smokers did not want to quit, they did not think that e-cigarettes would help them quit, and they thought they did not need help to quit. Since most smokers did not intend to quit or did not think e-cigarettes would help quitting, it was not surprising that e-cigarette cost was not a factor for most smokers.

Although the evidence on the health risks of e-cigarettes and their potential effectiveness as a cessation tool is growing rapidly, the long-term health risks of e-cigarette use is still unknown^4^. In addition, it is still not entirely clear how the shortterm health risks of e-cigarettes compare with those of combustible cigarettes^4^. Furthermore, the evidence on the effectiveness of e-cigarettes as a cessation tool is equivocal^10,11,31^. Based on the comprehensive review by the National Academies of Sciences, Engineering, and Medicine (NASEM), e-cigarette aerosol contains fewer and lower levels of most toxicants emitted by combustible cigarettes^4,6^. However, toxicity is not the only factor to assess the relative health risks of e-cigarettes^32^. Given the wide variety of e-cigarette products on the market, more evidence is needed to evaluate the relative harm between e-cigarettes and combustible cigarettes, particularly for long-term users^32^. Although the most recent Cochrane literature review concluded that there was ‘moderate-certainty evidence’ that smokers using e-cigarettes with nicotine had higher quit rates compared to nicotine replacement therapy (NRT) products^31^, e-cigarettes have not been approved by the FDA as a smoking cessation tool^29^. The World Health Organization (WHO) also concluded that the potential of e-cigarettes as a cessation aid is still unclear and the safest approach is to use neither cigarettes nor e-cigarettes^32^.

These uncertainties associated with e-cigarettes provide important context to understand why it is important to examine the factors explaining the low rates of e-cigarette use among Chinese smokers. This information can help us make predictions about the future trends of cigarette smoking and e-cigarette use in China. Our study results revealed that a significant proportion of Chinese smokers believed e-cigarettes were linked with smoking cessation. Chinese smokers’ perception of the potential role of e-cigarettes in aiding cessation was likely the result of exposure to e-cigarette advertising, which often contained unsubstantiated claims about the e-cigarette’s effectiveness in helping smokers quit and its role as a better and healthier substitute for cigarettes^17,33,34^. As China increases its effort to promote smoking cessation^35,36^, it is expected that more Chinese smokers will seek to quit smoking, and some of these smokers may start to use e-cigarettes as a result, similar to what has been observed in the US and UK. As such, policies that encourage smokers to quit and discourage e-cigarette use will be needed if future studies demonstrate that e-cigarettes are harmful and that they are not effective in helping smokers quit. On the contrary, policies that encourage smokers to switch to e-cigarettes can be adopted if future studies can demonstrate that e-cigarettes are effective in helping smokers quit.

To date, the best way to encourage smokers to quit is still the evidence-based smoking cessation methods, which include professional counselling, prescribed stop smoking drugs, and prescribed and over-the-counter nicotine replacement therapy (NRT) products, such as nicotine gum, patch, lozenge, spray, etc.^37,38^. Previous studies indicate that smokers who had formal cessation assistance were more likely to quit successfully than smokers without any formal assistance^38,39^. However, smoking cessation services are still very limited in China^40^. The 2018 China GATS reported that more than 90% of quit attempts made in the last 12 months were without any cessation aid^14^. To reduce smoking in China, it is important to provide evidence-based smoking cessation services to Chinese smokers.

Another contribution of our study was the analysis across population subgroups, which previous studies did not examine. In our study, the estimated adjusted associations between reasons for never having tried e-cigarettes and sociodemographic characteristics showed that, in general, there were no statistically significant differences among subgroups, except that government employees, teachers and healthcare providers were less likely to select ‘I do not want to quit smoking’. This may be due to the fact that smoking is usually prohibited in government buildings, schools, and hospitals in China, therefore people working in those occupations may have higher intention to quit^12^. In addition, factory, business, and service industry employees were less likely to select ‘I do not think they would help me to quit or cut down’, indicating a more positive view of e-cigarettes as a cessation tool among workers in those industries. This may be due, in part, to the high likelihood of exposure to misinformation about e-cigarettes’ role in smoking cessation via peers and targeted e-cigarette marketing among workers in factories and the services industry^41,42^. The lack of differences in reporting reasons or concerns for not trying e-cigarettes among population subgroups may be due to the fact that e-cigarettes were a relatively new product in China, and smokers with different demographic characteristics may share similar, yet limited, knowledge and opinions towards this product. Given the rapid changes in the tobacco market in China, continued monitoring of the e-cigarette market and use of e-cigarettes is critical for guiding potential future tobacco control policies and interventions that target e-cigarettes.

### Limitations

Our study has some limitations. We did not ask reasons for discontinuing the use of e-cigarettes among smokers who have tried but stop using e-cigarettes. About 18% of smokers reported ever use of e-cigarettes and about 4% of smokers were current e-cigarette users. This means that about 14% of smokers discontinued using e-cigarettes. Studies in the US found that more than one-third of dual users of e-cigarettes and combustible cigarettes discontinued using e-cigarettes while continuing smoking combustible cigarettes^43^, and the most commonly reported reasons for not continuing using e-cigarettes included: ‘They did not feel like smoking a cigarette’, ‘They did not help me deal with cravings for smoking’, ‘I only ever tried them to see what they were like’ and ‘They cost too much’^9,21,44^. Similar findings were reported among UK smokers who discontinued using e-cigarettes^1,45^. Due to this data limitation, we were unable to compare the differences in reasons for not using e-cigarettes between smokers who had never tried e-cigarettes and smokers who discontinued using e-cigarettes. Other limitations include: data used in the study came from four citywide representative surveys, so our findings may not be generalized to other Chinese cities or to the rural areas in China. Additionally, smoking status and reasons for never having tried e-cigarettes were self-reported, which may introduce a social desirability bias. Finally, we did not include an open-ended response category for the question asking reasons for never having tried e-cigarettes among Chinese smokers, therefore there might be other reasons not captured by our measure.

## CONCLUSIONS

Our study found that the top reasons that a vast majority of smokers, albeit being aware of e-cigarettes, had never tried them were low intention to quit smoking, concerns about e-cigarettes’ effectiveness as a smoking cessation method and belief of being able to quit any time without aid. More efforts are needed to promote smoking cessation in China. Given the current uncertainties associated with the health risks of e-cigarettes and their potential role as smoking cessation aids, it is important to promote cessation methods that have already proven to be safe and effective. Targeted health education campaigns towards smokers who use e-cigarettes are needed to accurately communicate the potential health risks of e-cigarettes.
